# Hydrolysis of the Borohydride Anion BH_4_^−^: A ^11^B NMR Study Showing the Formation of Short-Living Reaction Intermediates including BH_3_OH^−^

**DOI:** 10.3390/molecules27061975

**Published:** 2022-03-18

**Authors:** Eddy Petit, Fabrice Salles, Damien Alligier, Umit B. Demirci

**Affiliations:** 1 Institut Européen des Membranes, IEM—UMR 5635, ENSCM, CNRS, University Montpellier, 34095 Montpellier, France; eddy.petit@umontpellier.fr (E.P.); alligier.damien@gmail.com (D.A.); 2 ICGM, University Montpellier, CNRS, ENSCM, Montpellier, France; fabrice.salles@umontpellier.fr

**Keywords:** borate, borohydride, hydrogen, hydrolysis, short-living intermediates

## Abstract

In hydrolysis and electro-oxidation of the borohydride anion BH_4_^−^, key reactions in the field of energy, one critical short-living intermediate is BH_3_OH^−^. When water was used as both solvent and reactant, only BH_3_OH^−^ is detected by ^11^B NMR. By moving away from such conditions and using DMF as solvent and water as reactant in excess, four ^11^B NMR quartets were observed. These signals were due to BH_3_-based intermediates as suggested by theoretical calculations; they were DMF·BH_3_, BH_3_OH^−^, and B_2_H_7_^−^ (i.e., [H_3_B−H−BH_3_]^−^ or [H_4_B−BH_3_]^−^). Our results shed light on the importance of BH_3_ stemming from BH_4_^−^ and on its capacity as Lewis acid to interact with Lewis bases such as DMF, OH^−^, and BH_4_^−^. These findings are important for a better understanding at the molecular level of hydrolysis of BH_4_^−^ and production of impurities in boranes synthesis.

## 1. Introduction

Sodium borohydride NaBH_4_ in alkaline aqueous solution is a potential fuel of low-temperature fuel cell [1]. It is regarded as an *indirect* fuel (i.e., H carrier) when it is hydrolyzed to produce H_2_, the as-produced H_2_ then feeding a fuel cell (Equation (1)) [2]. It is regarded as a *direct* fuel (i.e., reductant) when it directly feeds a direct liquid fuel cell to be electro-oxidized (Equation (2)) [3]:

BH_4_^−^ (aq) + 4H_2_O (l) → B(OH)_4_^−^ (aq) + 4H_2_ (g) (1)

BH_4_^−^ (aq) + 8OH^−^ (l) → B(OH)_4_^−^ (aq) + 4H_2_O (l) + 8e^−^ (2)

The aqueous solution has to be alkaline, that is, stabilized [4], because this is the only way to prevent spontaneous (exothermic, with an enthalpy of about −240 kJ mol^−1^ [5]) hydrolysis of BH_4_^−^ from occurring extensively. In hydrolysis (Equation (1)), a metal catalyst is therefore required to accelerate the production of H_2_ [6]. In electro-oxidation (Equation (2)), a metal electro-catalyst is required to promote the generation of a maximum of electrons (out of eight) [4]. However, the electro-catalyst also acts as catalyst of hydrolysis, a reaction that is in this case regarded as heterogeneous because it is detrimental to the fuel cell faradaic efficiency [7].

In hydrolysis (Equation (1)) as well as in electro-oxidation (Equation (2)), complete reaction implies transformation of BH_4_^−^ into B(OH)_4_^−^ via formation of short-living intermediates. For spontaneous hydrolysis, Mochalov et al., suggested in 1965 BH_3_OH^−^, BH_2_(OH)_2_^−^, and BH(OH)_3_^−^ as possible short-living intermediates [8]. They showed, for instance, that the direct transformation of BH_4_^−^ into B(OH)_4_^−^ has the same kinetic constant (*k* = 5.31 × 10^7^ min^−1^) as the transformation of BH_4_^−^ into BH_3_OH^−^ (*k*’ = 5.15 × 10^7^ min^−1^). The same year, Gardiner and Collat suggested the formation of BH_3_, BH_3_OH^−^, and [H]^+^[BHOH]^−^ as possible short-living intermediates [9]. More recently, Guella et al. reported that, by ^11^B nuclear magnetic resonance (NMR) spectroscopy, they detected only BH_4_^−^ and B(OH)_4_^−^ (Equation (1)) for a Pd-catalyzed hydrolysis [10]. The non-detection of other species was explained by the fact that the hydrolysis intermediates are excessively short-living in their experimental conditions. By quantum chemical calculations, Lu et al. [11] confirmed Guella et al.’s explanation and modelled a multistep process involving the following hypothetical short-living intermediates (Equation (3)):

BH_4_^−^ → BH_3_OH^−^ → H_2_BO^−^ → BH_2_(OH)_2_^−^ → HB(O)OH^−^ → BH(OH)_3_^−^ → B(O)(OH)_2_^−^ → B(OH)_4_^−^ (3)

Comparable predictions were reported by Zhou et al. [12], Andrieux et al. [13], Churikov et al. [14], and Choi et al. [15] detected traces of BH_3_OH^−^ by using ^11^B NMR spectroscopy. It is therefore arguable whether BH_3_OH^−^, as the first short-living intermediate, directly hydrolyzes into B(OH)_4_^−^. This is a possible parallel pathway as suggested by Mochalov et al. [8] for example.

Budroni et al. [16] refers to BH_3_OH^−^ as a critical short-living intermediate. As discussed above, this applies to the hydrolysis reaction (Equation (1)). Interestingly, this also applies to electro-oxidation of BH_4_^−^ (Equation (2)) on metal electrodes (e.g., Pd, Pt, and Au) [17,18,19,20,21,22,23]. For instance, Molina Concha et al. [24] studied Pt-catalyzed electro-oxidation of BH_4_^−^ by in situ Fourier Transform Infrared (FTIR) spectroscopy. They observed that: (i) BH_3_OH^−^ formed at low potentials (<0.7 V) by hydrolysis of BH_4_^−^ and/or partial oxidation of BH_4_^−^; (ii) BH_3_OH^−^ quickly electro-oxidized into BH_2_ intermediates such as BH_2_OH and BH_2_(OH)_2_^−^; and (iii) the BH_2_ intermediates electro-oxidized into BO_2_^−^ at high potentials (>0.7 V).

Similarly, Nanayakkara et al. [25] investigated the mechanism of H_2_ release of BH_3_ in water and the following solvent effects by using MP2 quantum calculations. One H_2_O molecule interacting with BH_3_ led to an activation energy equal to 24.9 kcal mol^−1^, while the energy values ranged from 29 and 32 kcal mol^−1^ when one H_2_O molecule interacted with BH_3_ and another H_2_O molecule interacted with the H_2_O molecule bonded to BH_3_. The resulting enthalpy was estimated at 20 kcal mol^−1^ for the first configuration and ranged between 12 and 14 kcal mol^−1^ for the others.

The present study is to be seen against the background described above. Based on a systematic study using ^11^B NMR spectroscopy, we attempted to detect and identify any short-living intermediates in order to gain insight and better understanding of both hydrolysis and electro-oxidation of BH_4_^−^. Furthermore, theoretical investigations were performed for obtaining vibrational results, determining the sensitive frequencies and estimating the energies of the different hypothetical molecular structures.

## 2. Results and Discussion

### 2.1. Hydrolysis Conditions Where H_2_O Acts as Both Reactant and Solvent

In hydrolysis and electro-oxidation conditions, the fuel is an alkaline aqueous solution of BH_4_^−^ for which the concentration of BH_4_^−^ is usually kept low (typically < 1 M). We therefore set our experimental conditions to be in line with such practices: the concentration of NaOH was fixed as 0.1 M and the concentration of BH_4_^−^ (from NaBH_4_) was chosen as 0.66 M.

In hydrolysis and electro-oxidation conditions, the reaction is catalyzed by a metal catalyst and an electro-catalyst, respectively. We selected three bulk metals such as Pd, Pt, and Au (each as a piece of metal wire). They were selected because each has been used in hydrolysis [26] and electro-oxidation [22].

**Figure 1:**
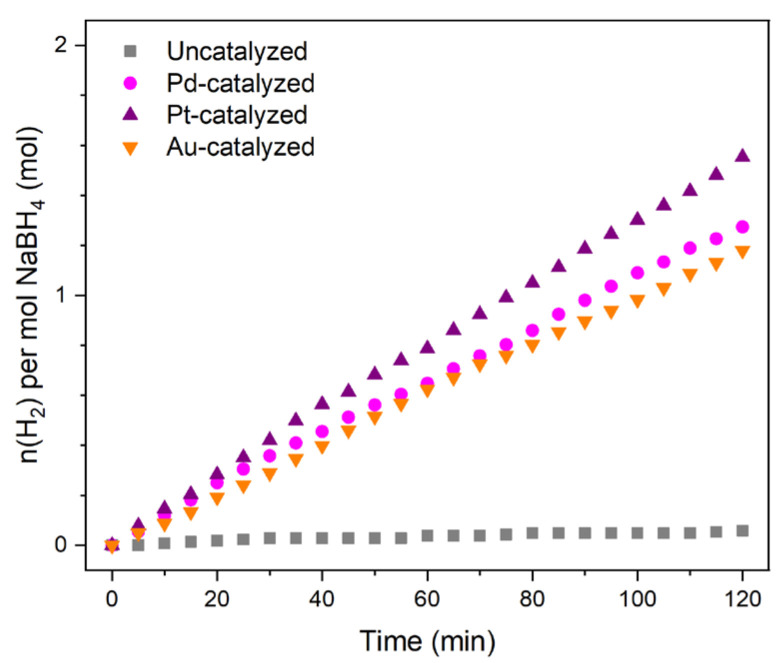
H_2_ evolution curve for the hydrolysis of 2 mL of an aqueous alkaline (0.1 M NaOH) solution of BH_4_^−^ (0.66 M; i.e., 50 mg NaBH_4_ in 2 mL) catalyzed by 16.1 mg of Pd, 14.5 mg of Pt, or 14.3 mg of Au at 30 °C. The H_2_ evolution curve for the uncatalyzed hydrolysis test is also shown. The *y*-axis has been limited to the range 0–2 for clarity.

In the present study and unlike in common practices [26], our objective was not to develop an active (or very active) hydrolysis catalyst. Our objective was to work with a lowly active catalyst so that the kinetics of H_2_ production remains slow when analyzing the solutions by ^11^B NMR spectroscopy. We thus focused on metals in bulk state, which is a state that offers the desired catalytic activity. We ensured this by performing a series of hydrolysis experiments. Typically, 2 mL of the aforementioned alkaline solution of BH_4_^−^ (corresponding to 50 mg of NaBH_4_) were put into contact with 16 mg of Pd, 14.5 mg of Pt, or 14.3 mg of Au at 30 °C. Regardless of the nature of the metal, it took 2 h to produce <1.6 mol H_2_ per mol BH_4_^−^ (Figure 1), that is, <53 mL H_2_ (out of 132 mL for a conversion of 100%). This means a H_2_ generation rate of <0.45 mL(H_2_) min^−1^ that is in agreement with our need. We also ensured that, in the absence of any metal, the alkaline solution of BH_4_^−^ was quite stable. At 30 °C, <0.1 mol H_2_ per mol BH_4_^−^ was produced in 2 h (namely, <3 mL(H_2_)).

**Figure 2:**
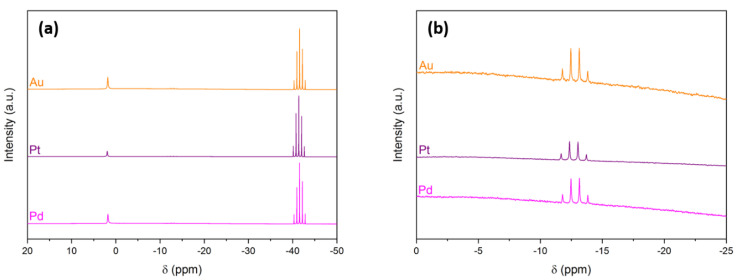
^11^B NMR spectra of the aqueous alkaline (0.1 M NaOH) solution of BH_4_^−^ (0.66 M; i.e., 50 mg NaBH_4_ in 2 mL) upon H_2_ evolution catalyzed by Pd, Pt, or Au at 30 °C. (**a**) Range between δ +20 and δ −50 ppm. (**b**) Focus on the range between δ 0 and −25 ppm.

The hydrolysis tests were repeated to analyze the solution by ^11^B NMR spectroscopy every hour. Similar to a previous study [13], we detected only three signals (examples of spectra in Figure 2; Table 1). The first main signal was a quintet at δ −41.5 ppm due to BH_4_^−^. The second main signal was a singlet at δ +1.9 ppm evidencing the formation of B(OH)_4_^−^ (Equation (1)). There was an additional minor and almost negligible signal, a quartet at δ −12.8 ppm. It was ascribed to the short-living intermediate BH_3_OH^−^ [10,15,27].

No additional ^11^B NMR signals that would be attributed to other short-living intermediates were seen. This might be explained by concentrations that are below the detection limit (ca. 1 × 10^−3^ mol L^−1^) of the spectrometer. This might be also explained by low symmetry of the intermediates’ structures, which would lead to broad signals of very low intensity and thus indistinguishable from the base line. It is worth mentioning that we used Gaussian 09 software to perform geometry optimization and NMR calculations for a series of possible intermediates including BH_2_(OH)_2_^−^ and BH(OH)_3_^−^. We found that the signals of BH_2_(OH)_2_^−^ and BH(OH)_3_^−^ should be a triplet and a doublet appearing between δ −7 and δ 0 ppm, respectively.

Another possible explanation of the absence of additional ^11^B NMR signals is that the experimental conditions were not suitable for detecting intermediates with a lifetime that is shorter than that of the detected BH_3_OH^−^. Based on our observations, we can state that the lifetime scale of BH_3_OH^−^ is of tens of seconds, whereas it might be much shorter (e.g., microseconds scale) for the other intermediates. Yet, the hydrolysis tests described above were performed in the presence of an excess of water: we used 2 mL (mol ratio H_2_O/BH_4_^−^ of 84) whereas about 0.1 mL (mol ratio H_2_O/BH_4_^−^ of 4) would be enough to totally hydrolyze BH_4_^−^. Water acted as both reactant and solvent, and the excess of water could be a favorable context to promote extremely fast hydrolysis of short-living intermediates.

### 2.2. Hydrolysis Conditions Where H_2_O Is Only a Reactant

In order to move away from the conditions using water as both reactant and solvent, we drew on two ancient reports dealing with hydrolysis of BH_4_^−^. Modler and Kreevoy investigated the hydrolysis of BH_4_^−^ (0.002 M) in moist acetonitrile (i.e., containing 0.6 M H_2_O) [28], and Taub et al. used aqueous dimethylformamide (DMF) [29]. We thus selected DMF as aprotic solvent of NaBH_4_ and used H_2_O as reactant only.

We prepared four 10 mL DMF solutions of BH_4_^−^ by dissolving 0.5 g of NaBH_4_ (1.32 M). A piece of the aforementioned Pd, Pt, and Au was added in each of three of the DMF solutions. The fourth DMF solution was kept metal-free and is denoted uncatalyzed. We then added 0.95 mL of alkaline (0.1 M NaOH) aqueous solution to each of the four DMF solutions (resulting in a concentration of H_2_O in DMF of 5.291 M). In these conditions, the mol ratio H_2_O/BH_4_^−^ was about four as for the stoichiometric hydrolysis reaction (Equation (1)). The as-prepared solutions were analyzed by ^11^B NMR spectroscopy. It is worth mentioning that in such conditions, the hydrolysis was expected to be slow. Accordingly, the solutions were analyzed every 24 h for 3 days.

The ^11^B NMR spectra focusing on the δ range varying from +20 to −50 ppm (Figure S1) showed only the quintet at δ −39.7 ppm due to BH_4_^−^. By zooming over the δ range varying from +20 to −30 ppm (Figure S2), it was possible to distinguish an additional signal of very small intensity at δ −14.1 ppm, namely the quartet due to BH_3_OH^−^. The quartet could be seen after 24 h for the Pd-, Pt-, and Au-catalyzed solutions, and after 48 h for the uncatalyzed solution. These results highlighted that, in the stoichiometric conditions, the hydrolysis took place to a negligible extent. Another observation is that, even in the absence of a metal, hydrolysis spontaneously took place. The non-detection of B(OH)_4_^−^ may have up to three explanations: the amount of H_2_O was too low and the H_2_O molecules were very diluted in DMF, which hindered interaction-reaction with BH_4_^−^ and BH_3_OH^−^; borates including B(OH)_4_^−^ were practically insoluble in DMF [30] and may have precipitated; and/or, the concentration of B(OH)_4_^−^ was below the detection limit.

### 2.3. Hydrolysis Conditions Where H_2_O Is a Reactant in Excess

We therefore repeated the experiments while increasing the water content: the mol ratio H_2_O/BH_4_^−^ passed from 4 to 32. Once more, we prepared four 10 mL DMF solutions of BH_4_^−^ (1.32 M) and added 7.6 mL of alkaline (0.1 M NaOH) aqueous solution. In comparison to the experiments presented in Section 2.1, the present series used water to a lesser extent (i.e., mol ratio H_2_O/BH_4_^−^ of 32 versus 84) and the 32 equivalents of H_2_O were dispersed in 10 mL of DMF, mitigating the hydrolysis of BH_4_^−^.

**Figure 3:**
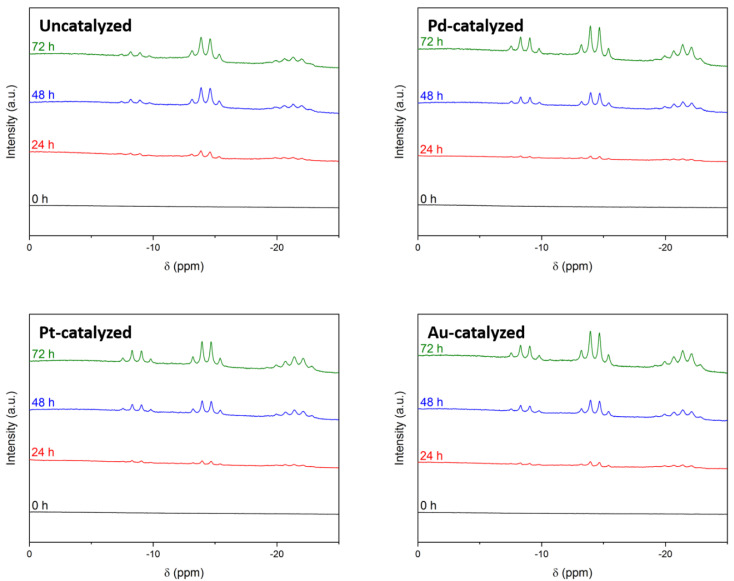
^11^B NMR spectra of the 10 mL DMF solutions of BH_4_^−^ (1.32 M) hydrolyzed by 7.6 mL of alkaline (0.1 M NaOH), uncatalyzed or catalyzed by Pd, Pt, or Au after 0, 24, 48 and 72 h. These spectra focus on the range between δ 0 ppm and δ −25 ppm to show the signals at δ < 0 ppm.

As before, the ^11^B NMR spectra (Figure S3) mainly showed the quintet at δ −40.5 ppm due to BH_4_^−^, and B(OH)_4_^−^ was not observed because of the reasons listed at the end of the previous section. In contrast to the results discussed above, the ^11^B NMR spectra showed additional signals at δ < 0 (Figure 3). This is discussed hereafter.

The first of the additional signals was a quartet at δ −14.4 ppm. As for our experiments discussed above, it was ascribed to BH_3_OH^−^.

The second of the additional signals was also a quartet, centered at δ −8.9 ppm. It indicated the formation of another BH_3_-containing intermediate.

**Figure 4:**
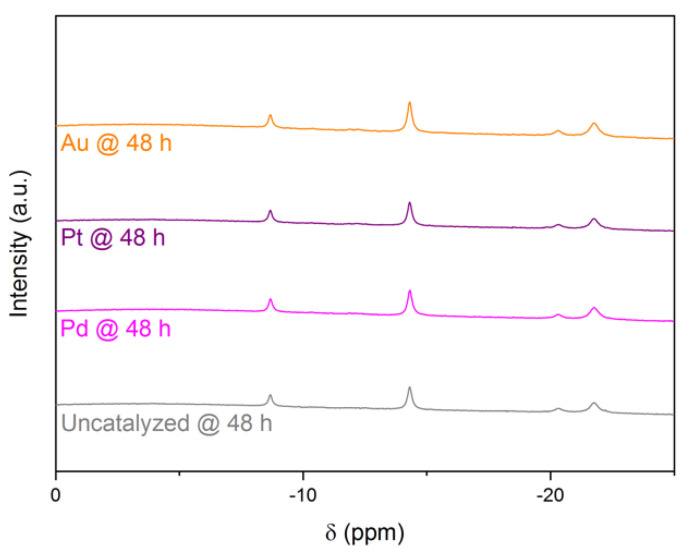
Proton-decoupled ^11^B NMR of the 10 mL DMF solutions of BH_4_^−^ (1.32 M) hydrolyzed by 7.6 mL of alkaline (0.1 M NaOH), uncatalyzed or catalyzed by Pd, Pt, or Au after 72 h.

The third of the additional signals appeared as a multiplet located between δ −18.5 ppm and δ −23.5 ppm. With the help of ^1^H-decoupled ^11^B NMR spectroscopy, we shed light on its nature. It was the result of two distinct signals peaking at δ −20.3 ppm and δ −21.8 ppm (Figure 4). By deconvolution of the signal, we found that the two signals were more likely to be two overlapping quartets, thereby indicating the formation of two other BH_3_ intermediates (Figure S4 and Table S1).

To summarize the above: the hydrolysis of DMF-solubilized BH_4_^−^ in the presence of 32 equivalents of H_2_O involved more intermediates than the only short-living intermediate BH_3_OH^−^. There were three additional intermediates and they all showed a quartet in ^11^B NMR spectroscopy, indicating that they all were made up of the BH_3_ group.

**Figure 5:**
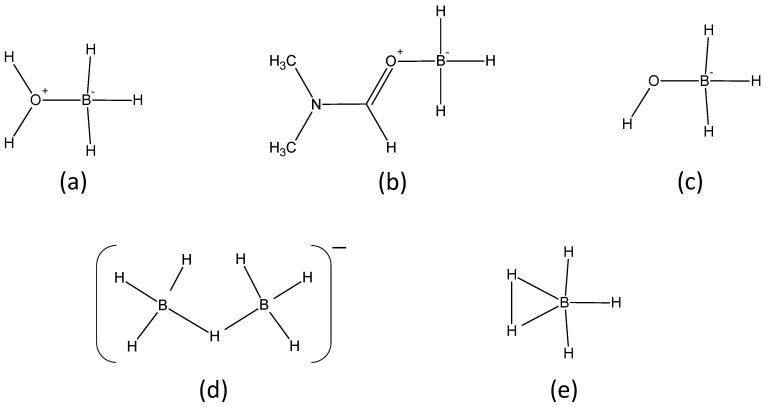
Possible BH_3_ intermediates showing a quartet in ^11^B NMR: (**a**) H_2_O·BH_3_, (**b**) DMF·BH_3_, (**c**) BH_3_OH^−^, (**d**) B_2_H_7_^−^, and (**e**) BH_3_(H_2_).

We therefore focused our efforts on attributing the aforementioned quartets to possible BH_3_ intermediates. We thought about any species likely to form in our conditions while exploring the open literature [31,32,33,34]. The following ones were listed (Figure 5):

- The complex H_2_O·BH_3_ because H_2_O is a Lewis base able to complex the Lewis acid BH_3_;
- The complex DMF·BH_3_ because DMF is Lewis bases able to complex BH_3_;
- The anion BH_3_OH^−^;
- The anion B_2_H_7_^−^ (i.e., [H_3_B−H−BH_3_]^−^ or [H_4_B−BH_3_]^−^); and
- The pentacoordinate BH_3_(H_2_).

According to Tague and Andrews [34], the last species BH_3_(H_2_) possibly acts as intermediate before the formation of BH_3_OH^−^ by reaction of BH_4_^−^ and H_2_O.

We then used Gaussian 09 software to perform geometry optimization and NMR calculations for each of these possible intermediates. We found the chemical shifts listed in Table 2. As observed in this table, a relatively good agreement between CASTEP and Gaussian 09 results was obtained considering the two investigated functionals (B3LYP for Gaussian 09 and PBE for CASTEP), except for BH_3_(H_2_). In the case of this species, the impact of the dispersion could be invoked but additional calculations using DFT-D in CASTEP showed a very small influence of dispersion on the calculations. It is worth mentioning that in a previous study [31], the chemical shift of B_2_H_7_^−^ in THF as solvent was reported to be δ −26 ppm. Similarly, using CASTEP calculations, we found comparable values (Table 2). We also calculated the chemical shifts for the intermediates based on BH_4−x_(OH)_x_^−^ (with x = 1, 2, 3, 4), such as: BH_4_^−^ with δ −51.5 ppm; BH_3_OH^−^ with δ −11.4 ppm; BH_2_(OH)_2_^−^ with δ +0.1 ppm; BH(OH)_3_^−^ with δ +1.1 ppm; and B(OH)_4_^−^ with δ +3.1 ppm.

Going back to the results presented in Figure 3 and using the data in Table 2, we ascribed the quartets at follows. The signals at δ −8.9 ppm and δ −14.4 ppm (Figure 3) were unambiguously attributed to DMF·BH_3_ and BH_3_OH^−^. Because the calculated chemical shift of BH_3_(H_2_) is much different from that of remaining signals at around δ −21 ppm, we discarded its formation. We also discarded the formation of H_2_O·BH_3_ due to the absence of signals at around 0 ppm in our experimental conditions. Accordingly, the partly overlapping quartets are at δ −20.3 ppm and δ −21.8 ppm and are attributed to B_2_H_7_^−^ and B_2_H_7_^−^ in interaction with H_2_O. Indeed, the chemical shift for the quartet due to [B_2_H_7_·H_2_O]^−^ was calculated as −28.1 ppm using Gaussian 09 and −32.6 ppm using CASTEP; these shifts were close to those calculated for B_2_H_7_^−^ (Table 2).

**Figure 6:**
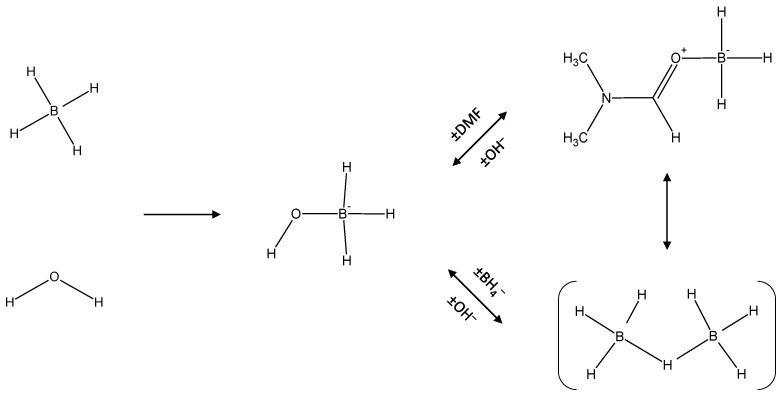
Mechanistic sequence illustrating the formation of the identified BH_3_-based intermediates BH_3_OH^−^, DMF·BH_3_, and B_2_H_7_^−^.

Based on the experimental results reported above and supported by the calculations performed, we suggest that the BH_4_^−^ anions dissolved in DMF are able to react with H_2_O taken in excess to form BH_3_-based intermediates such as DMF·BH_3_, BH_3_OH^−^, and B_2_H_7_^−^. These intermediates are much likely to be in equilibrium. Based on the discussions reported in [30], we thus suggest that in DMF, BH_3_OH^−^ forms first and DMF·BH_3_ and B_2_H_7_^−^ forms from BH_3_OH^−^ (by substitution of Lewis bases). This is illustrated in Figure 6.

## 3. Materials and Methods

Sodium borohydride NaBH_4_ (99%), sodium hydroxide NaOH (≥98%), *N*,*N*-dimethylformamide C_3_H_7_NO (DMF; 99.8%, anhydrous), Pt wire (99.9%, Ø 1.0 mm), Pd wire (99.9%, Ø 1.0 mm), and Au wire (99.95%, Ø 1.0 mm) all from Sigma-Aldrich were used as received. We stored and handled them in our argon-filled glove box (MBraun M200B, with O_2_/H_2_O < 0.1 ppm). We used Milli-Q deionized water (18.2 MΩ cm) and it was degassed by bubbling argon for 30 min before its use.

In a first step, the hydrolysis conditions were such that water acted as both reactant and solvent. The H_2_ evolution experiments were performed as follows. Under argon, 50 mg of NaBH_4_ were transferred in a Schlenk tube (used as hydrolysis reactor). For the catalyzed experiments, a piece of metal wire (16.1 mg of Pd, 14.5 mg of Pt, or 14.3 mg of Au) was also transferred in the tube. The tube was sealed and the glove box was taken out, installed to our hydrolysis set-up (reactor connected to an inverted burette via a cold trap kept at 0 °C), and immersed in an oil bath at 30 °C. The hydrolysis reaction was started by injecting 2 mL of an aqueous alkaline (0.1 M NaOH) solution. In these conditions, the mol ratio H_2_O/BH_4_^−^ was 84. The displacement of the blue-colored liquid in the inverted burette due to the generated H_2_ was video monitored. The H_2_ evolution experiments were repeated to analyze the solution by ^11^B NMR spectroscopy (Bruker Avance 400 NMR spectrometer equipped with a BBOF probe; BF_3_·OEt_2_ as reference; acetonitrile-d_3_ such as ≥99.8 atom % D and from Sigma-Aldrich).

In a second step, the hydrolysis conditions were modified such that water only acted as reactant. To do so, 10 mL of DMF was used as solvent of 50 mg of NaBH_4_. To this solution prepared under argon, a piece of metal was added to catalyze the reaction. The hydrolysis reaction was started by injecting 0.95 mL of alkaline (0.1 M NaOH) aqueous solution. The concentration of H_2_O in DMF was 5.291 M and the mol ratio H_2_O/BH_4_^−^ was about 4. The solutions were analyzed by ^11^B NMR spectroscopy every 24 h for 3 days.

In a third step, the hydrolysis conditions were once again modified. They were such that the water amount in DMF was increased and the mol ratio H_2_O/BH_4_^−^ passed from 4 to 32. Otherwise, the solutions were prepared similarly and they were analyzed by ^11^B NMR spectroscopy every 24 h for 3 days.

We finalized the attribution of the ^11^B NMR signals using theory and calculations. We used Gaussian 09 software to perform geometry optimization, vibrational analysis, and the NMR calculations. The molecular structures were determined by density functional theory calculations. A gas phase geometry optimization of the Gibbs free energy was calculated using B3LYP hybrid density functional with 6-311(++)G(2d,p) basis set at 298.15 K. NMR spectra (NMR references: TMS and BF_3_-OEt_2_) were predicted by using the same level of theory (B3LYP/6-311++G(2d,p)). Additional computational methods were used to probe the structural properties of the different intermediates (Figure 5 and Table 2). As the reactions are difficult to stop, to isolate the structures, molecular simulations appeared to be the most powerful strategy to determine the corresponding spectroscopic properties. In complement of Gaussian 09 calculations to determine the NMR chemical shifts, calculations consisting into geometry optimization and NMR properties determination were performed using CASTEP implemented in Materials Studio 2020 [35]. This is a DFT-based code using the projector-augmented waves (PAW) and gauge-included projector-augmented waves (GIPAW) algorithms for NMR chemical shifts, respectively. Here, the PBE functional was used in the generalized gradient approximation (GGA) for the exchange correlation energy. The core−valence interactions were described by norm-conserving pseudopotentials within the NMR CASTEP package and without implementation of any additional corrections. A kinetic energy cut-off was considered and the size of the box was fixed at 10 Å (additional calculations have been performed by considering a box size fixed at 20 Å and leading to similar results), which produced converged results for geometry optimization and NMR shielding determination. The convergence of the self-consistent field (SCF) calculations were reached when the total energy variation of the system was lower than 10^−5^ eV/atom, the maximum force variation was lower than 0.03 eV/Å, and the maximal displacement was lower than 0.001 Å. In order to compare the GIPAW calculated ^11^B shielding values with the corresponding experimental values, the following expression was used: *δ*_iso, calc_ = *σ*_ref_ − *σ*_iso_, where *σ*_ref_ corresponds to the value obtained for ^11^B (BH_3_-OEt_2_) and *σ*_iso_ is the computational value for the investigated species.

Additional calculations were performed with CASTEP to investigate the effect of the dispersion (by considering DFT-D corrections (suing OBS method implemented in Materials Studio)) and the use of ultrasoft pseudo-potentials. A small influence on the NMR properties was observed if the dispersion was taken into account, while the use of ultrasoft pseudo-potentials led to stronger variations.

## 4. Conclusions

When hydrolysis of BH_4_^−^ took place in water that acted as both solvent and reactant, only one short-living intermediate was detected. It was the well-known BH_3_OH^−^. In such conditions, the amount of water was excessive, offering a favorable environment to the complete hydrolysis of each BH_4_^−^ into B(OH)_4_^−^. When hydrolysis of BH_4_^−^ took place in DMF in the presence of a stoichiometric amount of water, only BH_3_OH^−^ was detected again. In these conditions, the amount of water was too low and, if any, the other intermediates were not detected because of too low concentrations (below the detection limit). When hydrolysis of BH_4_^−^ took place in DMF as solvent and in the presence of an excess of water, four BH_3_-based intermediates were detected, as evidenced by ^11^B NMR quartets peaking at δ −8.9, δ −14.4, δ −20.3, and δ −21.8 ppm. Using geometry optimization and calculations, these signals could be ascribed to DMF·BH_3_, BH_3_OH^−^, and B_2_H_7_^−^ (in two conformations or in interaction with DMF or H_2_O) that are likely to be equilibrium. This illustrates the capacity of the Lewis acid BH_3_ to interact with Lewis bases such as DMF, OH^−^, and BH_4_^−^. We also suggest that in DMF, BH_3_OH^−^ forms first and DMF·BH_3_ and B_2_H_7_^−^ forms from BH_3_OH^−^. These findings are important from a fundamental point of view for a better understanding of hydrolysis of BH_4_^−^ at the molecular level. These findings are also important for a better understanding of production of boron-based impurities in synthesis of boranes; boranes can be produced from BH_4_^−^ in an organic solvent like DMF that may contain traces of moisture.

## Figures and Tables

**Table 1:** Chemical shifts (δ, in ppm) of the signals observed for the following three experiments: (a) hydrolysis conditions where H_2_O acts as both reactant and solvent; (b) hydrolysis conditions where H_2_O is only a reactant and DMF is used as solvent; (c) hydrolysis conditions where DMF is the solvent and H_2_O is a reactant in excess. I_1_ to I_3_ indicates the intermediates associated to the quartets observed in the spectra collected for the experiment (c).

Experiment	B(OH)_4_^−^	I_1_	BH_3_OH^−^	I_2_	I_3_	BH_4_^−^
(a)	+1.9		−12.8			−41.5
(b)			−14.1			−39.7
(c)		−8.9	−14.4	−20.3	−21.8	−40.5

**Table 2:** Chemical shifts (δ, in ppm) for the hypothetic BH_3_-based short-living intermediates plus that of BH_4_^−^ for comparison as calculated using Gaussian 09 and CASTEP. The experimental values for the experiment (c) are recalled, where I_1_ is proposed to be DMF·BH_3_, and I_2_ to be B_2_H_7_^−^.

Calculation	H_2_O·BH_3_	DMF·BH_3_	BH_3_OH^−^	B_2_H_7_^−^	BH_3_(H_2_)	BH_4_^−^
Gaussian 09	0	−6.3	−16.8	−29.7	−40.2	−54.7
CASTEP	+2	−8.2	−11.4	−31.8	−48.1	−51.5
Experim. (c)		−8.9	−14.4	−20.3		−40.5

## Supplementary Materials

The following supporting information can be downloaded at: https://www.mdpi.com/article/10.3390/molecules27061975/s1. Figure S1: ^11^B NMR spectra of the 10 mL DMF solutions of BH_4_^−^ (1.32 M) hydrolyzed by *0.95* mL of alkaline (0.1 M NaOH), uncatalyzed or catalyzed by Pd, Pt, or Au after 0, 24, 48, and 72 h. These spectra focus on the range between δ +20 ppm and δ −50 ppm; Figure S2: ^11^B NMR spectra of the 10 mL DMF solutions of BH_4_^−^ (1.32 M) hydrolyzed by 0.95 mL of alkaline (0.1 M NaOH), uncatalyzed or catalyzed by Pd, Pt, or Au after 0, 24, 48, and 72 h. These spectra focus on the range between δ +20 ppm and δ −30 ppm; Figure S3: ^11^B NMR spectra of the 10 mL DMF solutions of BH_4_^−^ (1.32 M) hydrolyzed by 7.6 mL of alkaline (0.1 M NaOH), uncatalyzed or catalyzed by Pd, Pt, or Au after 0, 24, 48, and 72 h. These spectra focus on the range between δ +20 ppm and δ −50 ppm; Figure S4: Deconvolution of the multiplet located between δ −18.5 ppm and δ −23.5 ppm for the ^11^B NMR spectrum of the 10 mL DMF solutions of BH_4_^−^ (1.32 M) hydrolyzed by 7.6 mL of alkaline (0.1 M NaOH) and catalyzed by Au after 48 and 72 h; Table S1: Results of the deconvolution made for the signal shown in Figure S4. The chemical shifts, Pascal’s triangles, and convergences are shown; the mol files of the structures are presented in Figure 5.
