# Supplementary material for: Hydrolysis of the Borohydride Anion BH4−: A 11B NMR Study Showing the Formation of Short-Living Reaction Intermediates including BH3OH−
Source: Molecules. 2022 Mar 18;27(6):1975. doi: 10.3390/molecules27061975 (PMC8955758; doi:10.3390/molecules27061975)
Supplement: Supplementary file 1 [file molecules-27-01975-s001.zip › molecules-1602770-supplementary.pdf]

Supplementary File

Hydrolysis of the borohydride anion BH_4^-: A ^{11}B NMR study showing the formation of short-living reaction intermediates including BH_3OH^-

Eddy Petit [1], Fabrice Salles [2], Damien Alligier [1] and Umit B. Demirci [1,*]

[1] Institut Européen des Membranes, IEM – UMR 5635, ENSCM, CNRS, Univ Montpellier, Montpellier, France
[2] ICGM, Univ. Montpellier, CNRS, ENSCM, Montpellier, France
* Correspondence: Correspondence: umit.demirci@umontpellier.fr

Supplementary Materials

Page 2	**Figure S1**	^{11}B NMR spectra of the 10-mL DMF solutions of BH_4^- (1.32 M) **hydrolyzed by 0.95 mL of alkaline** (0.1 M NaOH), uncatalyzed or catalyzed by Pd or Pt or Au, after 0, 24, 48 and 72 h. These spectra focus on the range between δ +20 ppm and δ –50 ppm.
Page 3	**Figure S2**	. ^{11}B NMR spectra of the 10-mL DMF solutions of BH_4^- (1.32 M) **hydrolyzed by 0.95 mL of alkaline** (0.1 M NaOH), uncatalyzed or catalyzed by Pd or Pt or Au, after 0, 24, 48 and 72 h. These spectra focus on the range between δ +20 ppm and δ –30 ppm.
Page 4	**Figure S3**	^{11}B NMR spectra of the 10-mL DMF solutions of BH_4^- (1.32 M) **hydrolyzed by 7.6 mL of alkaline** (0.1 M NaOH), uncatalyzed or catalyzed by Pd or Pt or Au, after 0, 24, 48 and 72 h. These spectra focus on the range between δ +20 ppm and δ –50 ppm.
Page 5	**Figure S4**.	Deconvolution of the multiplet located between δ –18.5 ppm and δ –23.5 ppm for the ^{11}B NMR spectrum of the 10-mL DMF solutions of BH_4^- (1.32 M) hydrolyzed by 7.6 mL of alkaline (0.1 M NaOH) and catalyzed by Au, after 48 and 72 h.

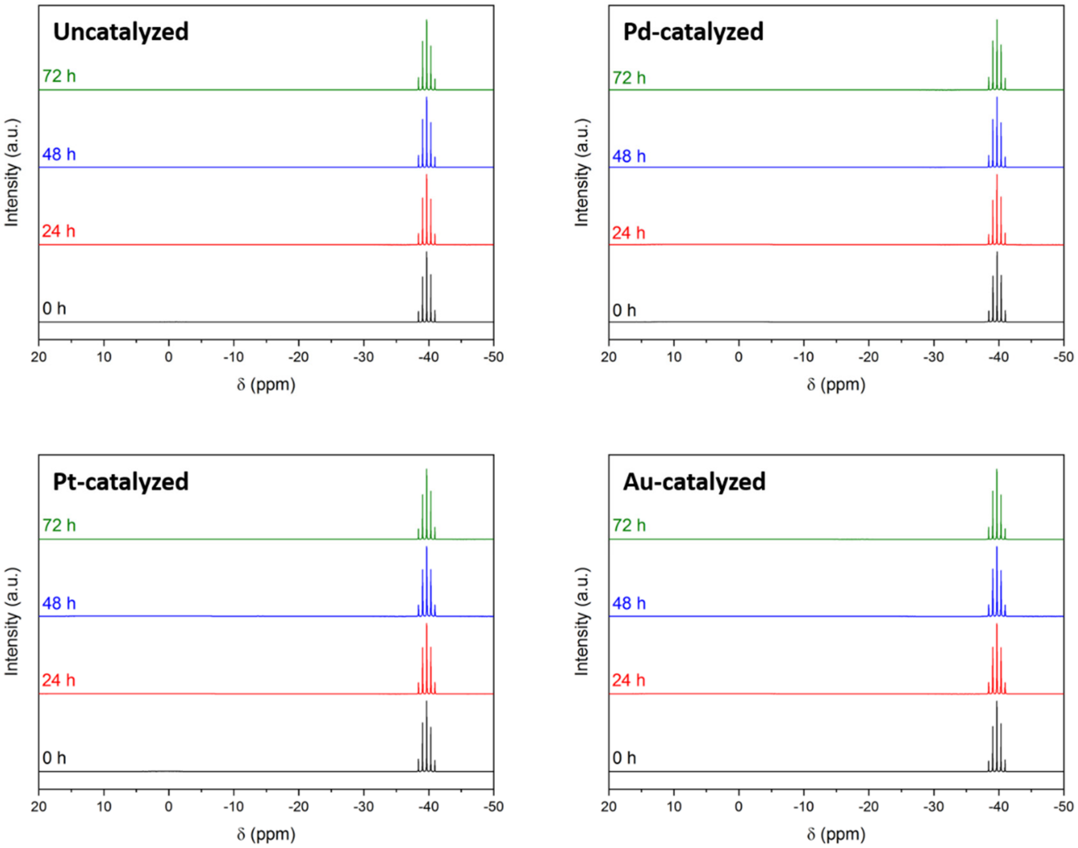

Figure S1. ^{11}B NMR spectra of the 10-mL DMF solutions of BH_4^- (1.32 M) hydrolyzed by 0.95 mL of alkaline (0.1 M NaOH), uncatalyzed or catalyzed by Pd or Pt or Au, after 0, 24, 48 and 72 h. These spectra focus on the range between δ +20 ppm and δ –50 ppm.

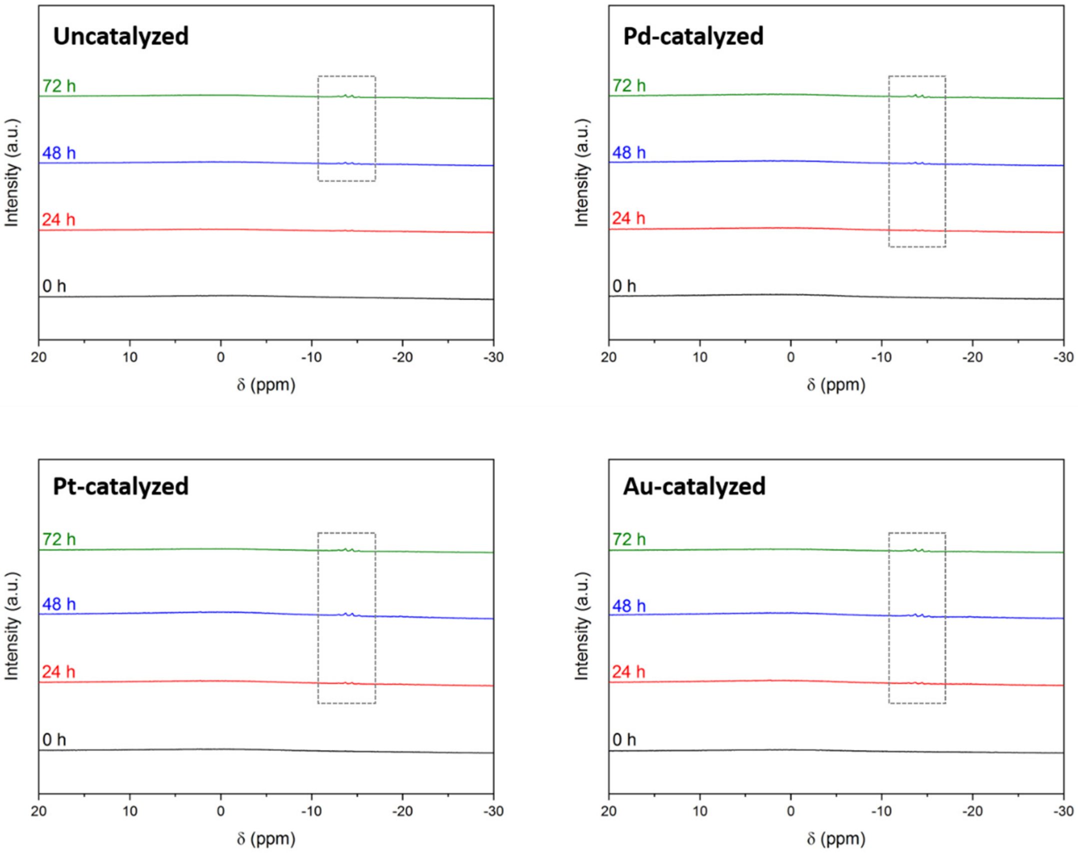

Figure S2. ^{11}B NMR spectra of the 10-mL DMF solutions of BH_4^- (1.32 M) hydrolyzed by 0.95 mL of alkaline (0.1 M NaOH), uncatalyzed or catalyzed by Pd or Pt or Au, after 0, 24, 48 and 72 h. These spectra focus on the range between δ +20 ppm and δ −30 ppm.

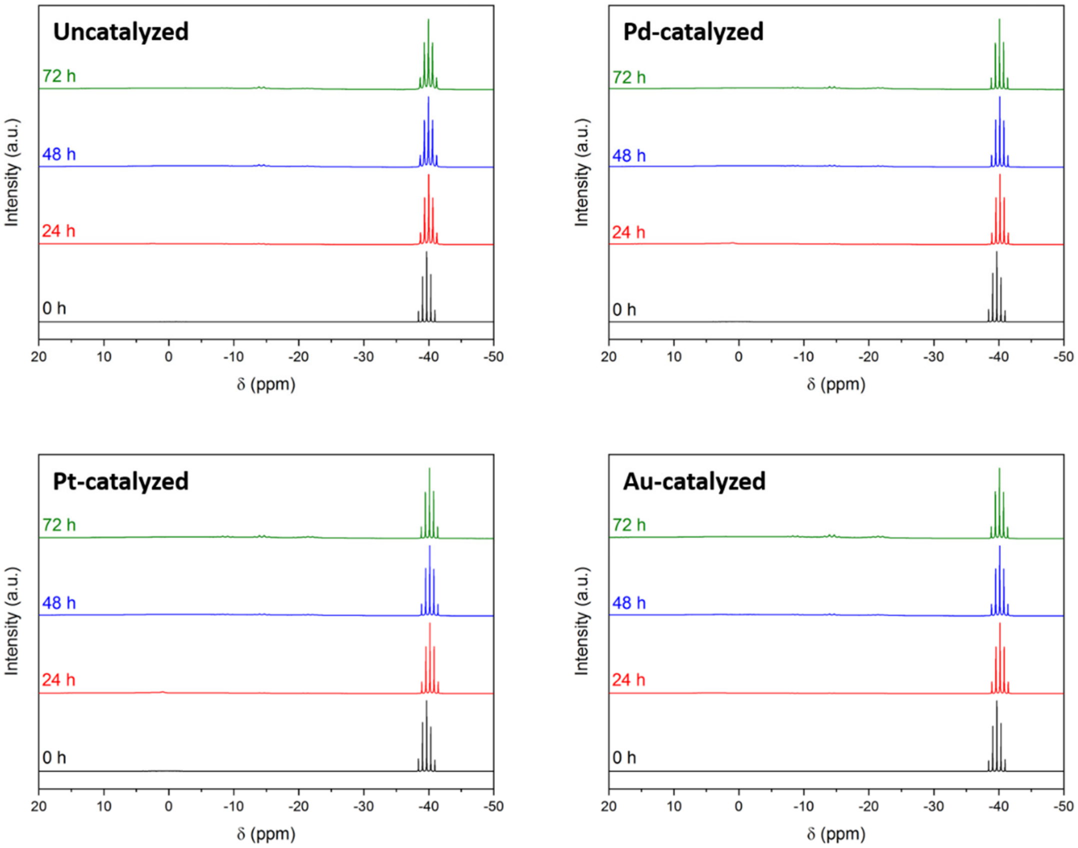

Figure S3. ^{11}B NMR spectra of the 10-mL DMF solutions of BH_4^- (1.32 M) hydrolyzed by 7.6 mL of alkaline (0.1 M NaOH), uncatalyzed or catalyzed by Pd or Pt or Au, after 0, 24, 48 and 72 h. These spectra focus on the range between δ +20 ppm and δ –50 ppm.

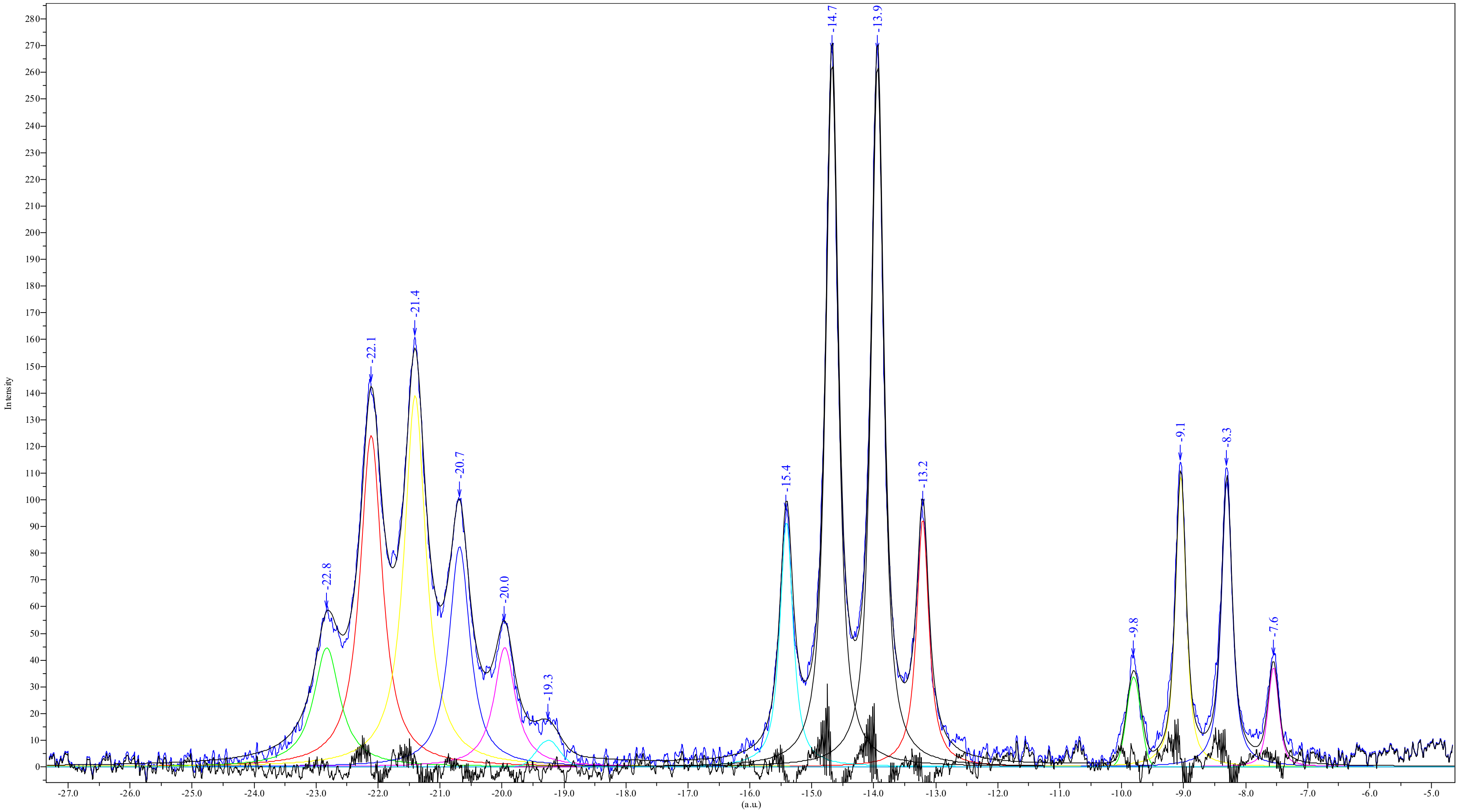

Figure S4. Deconvolution of the multiplet located between δ –18.5 ppm and δ –23.5 ppm for the ^{11}B NMR spectrum of the 10-mL DMF solutions of BH_4^- (1.32 M) hydrolyzed by 7.6 mL of alkaline (0.1 M NaOH) and catalyzed by Au, after 48 and 72 h.

Table S1. Results of the deconvolution made for the signal shown in Figure S4. The chemical shifts, the Pascal's triangles and the deviations versus the theoretical area are shown.

δ (ppm)	Pascal's triangle		Deviations (%)
−22.8334	1	0.09	−23
−22.1179	3	0.28	0
−21.4085	3 + 1	0.31	0
−20.6915	1 + 3	0.19	11
−19.9621	3	0.1	0
−19.2616	1	0.03	104
